# A qualitative analysis of decision-making among women with sexual violence-related pregnancies in conflict-affected eastern Democratic Republic of the Congo

**DOI:** 10.1186/s12884-018-1942-7

**Published:** 2018-08-08

**Authors:** Jennifer Scott, Monica A. Onyango, Gillian Burkhardt, Colleen Mullen, Shada Rouhani, Sadia Haider, Katherine Albutt, Ashley Greiner, Michael VanRooyen, Susan Bartels

**Affiliations:** 1 Department of Obstetrics & Gynecology, Beth Israel Deaconess Medical Center, 330 Brookline Avenue, Kirstein 3rd Floor, Boston, MA 02215 USA; 2 Division of Women’s Health, Brigham and Women’s Hospital, 75 Francis Street, Boston, MA 02115 USA; 3 Harvard Medical School, 25 Shattuck Street, Boston, MA 02115 USA; 4 Harvard Humanitarian Initiative, 14 Story Street, Cambridge, MA 02138 USA; 5 Department of Global Health, Boston University School of Public Health, 801 Massachusetts Avenue, Boston, MA 02118 USA; 6 Department of Obstetrics and Gynecology, Boston University School of Medicine, 85 East Concord Street, Boston, MA 02115 USA; 7 Department of Obstetrics and Gynecology, University of New Mexico, MSC 10 5582, Albuquerque, NM 87131 USA; 8 Department of Psychiatry, Boston Medical Center, One Boston Medical Center Place, Boston, MA 02118 USA; 9 Department of Emergency Medicine, Brigham and Women’s Hospital, 75 Francis Street, Boston, MA 02115 USA; 10 Department of Obstetrics & Gynecology, University of Chicago, 5837 S. Maryland Avenue, Chicago, IL 60615 USA; 11 Department of Surgery, Massachusetts General Hospital, 55 Fruit Street, Boston, MA 02114 USA; 12 Department of Emergency Medicine, Beth Israel Deaconess Medical Center, One Deaconess Road, Boston, MA 02215 USA; 13 Harvard School of Public Health, 677 Huntington Avenue, Boston, MA 02115 USA; 14 Department of Emergency Medicine, Queen’s University, 76 Stuart Street, Kingston, ON K7L 2V7 Canada

**Keywords:** Sexual violence, Pregnancy, Decision-making, Democratic Republic of the Congo, Conflict, Reproductive health services

## Abstract

**Background:**

Sexual violence is prevalent in conflict-affected settings and may result in sexual violence-related pregnancies (SVRPs). There are limited data on how women with SVRPs make decisions about pregnancy continuation or termination, especially in contexts with limited or restricted access to comprehensive reproductive health services.

**Methods:**

A qualitative study was conducted in Bukavu, Democratic Republic of the Congo (DRC) as part of a larger mixed methods study in 2012. Utilizing respondent-driven sampling (RDS), adult women who self-reported sexual violence and a resultant SVRP were enrolled into two study subgroups: 1) women currently raising a child from an SVRP (parenting group) and 2) women who terminated an SVRP (termination group). Trained female research assistants conducted semi-structured interviews with a subset of women in a private setting and responses were manually recorded. Interview notes were translated and uploaded to a qualitative software program, coded, and thematic content analysis was conducted.

**Results:**

A total of 55 women were interviewed: 38 in the parenting group and 17 in the termination group. There were a myriad of expressed attitudes, beliefs, and emotional responses toward SVRPs and the termination of SVRPs with three predominant influences on decision-making, including: 1) the biologic, ethnic, and social identities of the fetus and/or future child; 2) social reactions, including fear of social stigmatization and/or rejection; and 3) the power of religious beliefs and moral considerations on women’s autonomy in the decision-making process.

**Conclusion:**

Findings from women who continued and women who terminated SVRPs reveal the complexities of decision-making related to SVRPs, including the emotional reasoning and responses, and the social, moral, and religious dimensions of the decision-making processes. It is important to consider these multi-faceted influences on decision-making for women with SVRPs in conflict-affected settings in order to improve provision of health services and to offer useful insights for subsequent programmatic and policy decisions.

## Background

In conflict-affected settings, such as eastern Democratic Republic of the Congo (DRC), sexual violence is prevalent and impacts the sexual and reproductive health of survivors [1, 2]. An estimated 20% of female sexual violence survivors in eastern DRC have reported sexual violence-related pregnancies (SVRPs) [1–3]. There is increasing focus on addressing the unmet needs of women with SVRPs and children born from SVRPs, [1, 4, 5]; however, there are limited data on how to support women during their pregnancies, especially as they make important decisions about whether to continue or to terminate SVRPs.

Numerous factors influence decision-making regarding pregnancy continuation and pregnancy termination. Women consider their own needs, the potential child, existing children, their partner and other significant relations, and financial matters when making decisions about pregnancy termination [6]. Women also seek input from their social networks as part of the decision-making process, including partners, family members, and friends [7, 8]. Religious beliefs play an important role in decision-making, especially in the contexts of antenatal screening and fetal or maternal complications [9]. Furthermore, women contemplate pregnancy termination when the possibility of continuing the pregnancy is perceived to have adverse effects on their lives or on the lives of significant others [10].

For women with SVRPs, there are additional complexities and challenges that may influence decision-making. Decision-making for women with SVRPs is foremost done in the context of a pregnancy that has occurred without consent and resulted from violence. Women may process feelings of alienation from the fetus and their own bodies, as well as complex feelings about pregnancy termination [11]. Furthermore, as survivors of sexual violence, women with SVRPs may face barriers accessing health information and health services, due to fear of disclosure or stigma, which could also impact the process of informed decision-making and the timing of decisions related to pregnancy care [12–14]. In settings such as in DRC, legal restrictions on pregnancy termination and risks related to seeking pregnancy termination may also influence decision-making [15–17]. Pregnancy termination is highly restricted in DRC, even within the context of sexual violence [15, 18]. Technically, the DRC has signed and ratified the *Maputu Protocol,* which permits pregnancy terminations in cases of rape or incest. However, the 1982 DRC Penal Code stipulates that pregnancy terminations are illegal and subject to 5 - 15 years imprisonment [18].

The majority of studies on decision-making related to pregnancies are conducted among women without SVRPs and in settings where women have access to comprehensive reproductive health care, including prenatal care, family planning, and pregnancy termination services. Among the few studies that focus on decision-making among women with SVRPs, most of the evidence is derived from studies among women seeking pregnancy termination services. These studies often do not include the perspectives of women who may have contemplated pregnancy termination but who continued with an SVRP. There are also fewer data on the experiences of women with SVRPs in conflict-affected regions, and further data on decision-making in these contexts are needed to inform programs and policies.

We conducted a mixed methods study in 2012 in South Kivu Province, DRC, utilizing respondent-driven sampling (RDS) in order to understand the experiences and decision-making process of women with SVRPs [17, 19–24]. Previously published quantitative and qualitative findings from this study revealed that women with SVRPs had limited access to evidence-based pregnancy termination care and faced potential consequences from unsafe terminations [17, 22]. In brief, of women who terminated SVRPs in this study, most (11/17) used a traditional herb to terminate, others took quinine tablets (3/17), one terminated with an unspecified injection (1/17) and others used unspecified oral medications (2/17). This current paper presents an analysis of qualitative data to understand the influences on decision-making processes among women raising children born from SVRPs and among women who terminated SVRPs.

## Methods

### Study design

Using mixed quantitative and qualitative methods, this study assessed two subgroups of adult women with sexual violence-related pregnancies (SVRPs): 1) women currently raising children from SVRPs (parenting group) and 2) women who had terminated SVRPs (termination group). Data were collected in Bukavu, South Kivu Province, DRC in October–November 2012 using RDS. RDS is a peer-recruitment method used to sample hard-to-reach populations [25]. Detailed data on the implementation of RDS, quantitative data on mental health outcomes and parenting relationships from the parenting group, quantitative and qualitative data from the termination group, and qualitative data on disclosure patterns and psychosocial outcomes among both study groups were previously published [17, 19–24].

Adult women (18 years or older) who self-reported sexual violence and had a resultant SVRP between the start of the war in 1996 and the date of the study were eligible for participation. The parenting subgroup was comprised of women who had delivered a liveborn infant and were currently living with and caring for the child. The termination subgroup was comprised of women who reported termination of an SVRP. Women were ineligible for study participation if the pregnancy resulted in a spontaneous miscarriage or stillborn infant, if the child was not currently living with or in the care of the mother, or if the child was deceased.

In partnership with local organizations, initial participants were identified, interviewed, and given uniquely numbered coupons, which they used to recruit up to three peers who also met study criteria. Recruited peers who presented to the research office with a study coupon were screened for eligibility and if deemed eligible were interviewed, given uniquely numbered coupons, and then asked to recruit other potential participants. All eligible participants were invited to complete a quantitative survey and every 20th participant in the parenting group and every 5th participant in the termination group was invited to additionally complete a semi-structured qualitative interview. All participants were offered a headscarf (~$1 USD) as a token of appreciation and return transportation to the research office was reimbursed.

The Harvard School of Public Health’s institutional review board provided human subjects research approval. In lieu of formal review by a local ethics board in Bukavu, permission to conduct this research was granted to the research team by the medical inspector of South Kivu Province. Additionally, a community advisory board in Bukavu provided study oversight including input on participant compensation, reimbursement of transportation costs, and waiver of written consent. Verbal informed consent was obtained and interviews were conducted privately. Congolese female interviewers participated in a six-day training focused on research methods, ethical standards, and interviewing techniques immediately prior to data collection. At least one psychosocial assistant was present in the research office at all times to provide on-site counseling if needed. All participants received a referral card for medical and psychosocial care in Bukavu. No personal identifying information was collected and thus all data were anonymous from the beginning. The manually recorded interviews were stored securely in the study office and the electronic files were password protected. The study name and documentation did not disclose the sensitive nature of the research.

### Qualitative methodology

After obtaining verbal informed consent, interviewers verbally administered qualitative surveys in Kiswahili. Responses were manually recorded. The survey consisted of semi-structured questions to assess the context of the SVRPs and consequences related to the SVRPs including factors that may have influenced decision-making. Although the surveys were unique to each study subgroup, there were some similarities. For the parenting group, women were asked to describe the decision to continue the pregnancy and to raise the child, including what influenced the decision and whether they had considered terminating the pregnancy. For the termination group, women were asked how they came to a decision to end the pregnancy, including what may have influenced the decision and how they felt about the decision at the time of the study. Women in the termination group were also asked whether they considered continuing the pregnancy and if they could identify anything that would have made it easier for them to continue the pregnancy and raise the child.

The surveys were written in English, translated into Kiswahili by a translator in DRC and back translated by a different translator. Any translation differences were resolved by consensus with a third translator. A panel of local collaborators reviewed the translated questions for accuracy. A trained interpreter in DRC translated the interview notes into English and created electronic files.

### Qualitative data analysis

Electronic files were uploaded to the qualitative data analysis software, Dedoose (Version 5.0.11, Los Angeles, CA). Thematic content analysis was used to systematically code the data to identify themes and patterns [26, 27]. Both deductive and inductive approaches were employed [28, 29]. General categories were outlined based on quantitative data previously analyzed from this study and additional codes and categories were derived directly from the qualitative data.

Two researchers read the electronic interview notes line-by-line to identify relevant codes. Upon completion of this open coding, preliminary codes emerged and the two researchers independently coded all of the interview notes using these codes. Major themes and sub-themes were identified. Throughout coding, researchers triangulated the qualitative data with the previously analyzed quantitative results, peer debriefing, and existing literature. Constant comparative technique was applied, but the iterative process of concurrent data collection and analysis was not feasible due to study logistics. Coding inter-rater reliability, measured with a pooled Cohen’s kappa, was 0.92 [30].

## Results

Interviews were conducted with 55 women: 38 in the parenting group and 17 in the termination group. Among participants, the mean age was 33.7 years (18–60 years). Demographic data were incomplete for three participants. At the time of the study, 29% (16/55) of women were divorced or separated from their spouses, 24% (13/55) were widowed, 20% (11/55) were married, and 20% (11/55) were never married. Women were not asked about their marital status at the time of the SVRP. Most women in the study (45/52, 87%) had little or no formal education.

Overall, participants represented the predominant religious groups, Catholic 54% (28/52) and Protestant 46% (24/52) and the major ethnic group, Bashi 79% (41/52), in Bukavu. For participants with complete demographic data, more participants in the parenting group self-identified as being Catholic (21/36, 55%) compared to the termination group (7/16, 44%) but the difference was not statistically significant.

Participants expressed varied attitudes, beliefs, and emotional responses toward the SVRPs, which influenced their decision-making processes. Three predominant influences on decision-making about the SVRPs emerged from the data: 1) Women’s considerations of the identity of the pregnancy, fetus, and child in relation to the sexual assault and perpetrator, 2) Social influences on decision-making, including fear of social stigmatization and rejection, and 3) The influence of religious beliefs and moral considerations on women’s autonomy in the decision-making process.

### Theme 1: The identity of the pregnancy, fetus and child in relation to the sexual assault and perpetrator influenced decision-making about the SVRPs

A prominent theme emerged related to women’s concerns regarding the identity of the pregnancy, fetus, and future child in relation to the conception of a pregnancy after sexual violence. During the interviews, the majority of women described sexual assaults perpetrated by armed combatants, usually from a different ethnic group. Subsequently, the women expressed concerns about carrying a pregnancy resulting from violence, having a child fathered by an armed combatant or a child of mixed ethnicity. For some women, the fetus resulting from sexual violence was considered a “curse” or a “devil”. A few women expressed future hope for the child despite the circumstances.

*I couldn’t carry the pregnancy to term and deliver a child born from sexual violence. I thought he would behave like his father, the rapist.*

25 year-old woman, separated from her husband, who had terminated an SVRP.

*I didn’t want to mix children (Hutu children and Congolese children) and I didn’t want to have a child from an unknown father (or five fathers).*

40 year-old woman, married, who terminated an SVRP.

*I didn’t terminate the pregnancy but my concern was to have a child from a foreign ethnic group, a child fathered by a member of an armed group.*

38 year-old woman, separated from her spouse, raising a child from an SVRP.

*I was so happy to get it terminated and I felt stable. I felt as if I carried a little devil in my womb.*

60 year-old woman, husband missing, who had terminated an SVRP.

*I decided to carry it to term and raise the child among others though he was a burden. I could not raise my children separately and reject the one resulting from sexual violence because they have the same blood from me…I wanted to terminate it but I preferred to carry it to term and wish the child could bring comfort to lives…I knew that child was innocent. Once born he could live with others. He would be probably helpful and be so nice for me in the future.*

50 year-old woman, widowed, raising a child from an SVRP.

### Theme 2: Social influences on decision-making, including fear of social stigmatization and rejection

Women in both subgroups commonly described how anticipated attitudes from their spouses, families, and communities were important influences on decision-making, including fear of social stigmatization and rejection. Among married participants, the anticipated rejection and actual reaction of the spouse influenced decision-making, particularly among women who sought to terminate the SVRP. In many cases, women reported that decisions were made in order to protect and preserve marital and family relations. Some women, while reporting rejection from their spouses, had the support and acceptance of family, which influenced the decision to continue with the SVRP.

*I have no spouse any more. I live with my children…I was scared by the attitude of the community about such pregnancy. I didn’t will to carry that pregnancy to term.*

30 year-old woman, husband missing, who terminated an SVRP.

*I informed my spouse because we were living together...He was so angry that he asked me to learn from other women how to perform termination. I feel sad… I think to perform termination is a big mistake…. Basically, I was forced by my spouse to terminate. I agreed because I needed peace at home.*

27 year-old woman, married, who had terminated an SVRP.

*When we returned home in the village, my spouse rejected me...When my spouse rejected me, there was no need to perform termination. I decided to carry it to term and deliver it.*

25 year-old woman, separated from her husband, raising a child from an SVRP.

*I felt my life became hopeless. I felt very upset; my heart ached as I was rejected by my spouse when pregnant. I thought of performing termination but I changed my decision because I was not rejected by the family.*

38 year-old woman, separated from her husband, raising a child from an SVRP.

*I felt nervous. I felt as if I could commit suicide. But when I realized that I was not rejected by my family I decided to keep the pregnancy. I didn’t stop going to school. I decided to carry my pregnancy to full term.*

18 year-old woman, never married, raising a child born from an SVRP.

### Theme 3: The influence of religious beliefs and moral considerations on decision-making processes and women’s autonomy

References to religious beliefs and/or to “God” played an important role in women’s attitudes and beliefs toward the SVRP and in deciding whether to continue or to terminate the pregnancy, especially among women who identified as Catholic. Such references often revealed how a fear of God influenced decision-making, even in the context of a pregnancy conceived from sexual violence. Many women expressed that termination of an SVRP contradicted individual religious beliefs or those of their families and communities; however, there were women in the termination group who reported going against the beliefs of their church and terminating the pregnancy.

For women in the parenting subgroup, religious beliefs played an important role in their process of accepting the pregnancy conceived from sexual violence. Other women expressed that the pregnancy was God’s will and believed that the child from an SVRP might serve a particular purpose or might be a positive influence in life.

*I wanted to terminate the pregnancy but my aunt and the elder sister of my late mother advised me not to terminate it. They told me the child born from sexual violence was also created by God as any child. Then I decided to carry it to full term.*

35 year-old woman, separated from her spouse, raising a child from an SVRP.

*I decided to carry the pregnancy to term and then deliver it for fear of God. Definitely, only God knows about the future of the child. Hopefully he will make worth living for himself and for his mother.*

20 year-old woman, unmarried, raising a child from an SVRP.

*I carried the pregnancy to term because I was afraid of death and Christians are prohibited from performing termination. God could punish me if I killed his creature.*

60 year-old woman, widowed, raising a child from an SVRP.

Moral considerations were also expressed with references to what is considered “good” and “bad”, including references to perceptions of “sins” and consequences. There was discussion about how termination of SVRPs may be considered differently than the termination of other pregnancies that were not conceived from sexual violence. Within the moral frameworks, women also described preservation of dignity as an important element of decision-making and of processing the decisions that were made. The dilemma experienced by women during the decision-making process is evident in many of the interviews, with references to considering their own needs even if those needs differed from what was expected of them in their broader social contexts. Women expressed a spectrum of post-decision attitudes with some expressing regret after termination of the SVRPs and others experiencing a sense of relief and peace related to their decision.

*I think to terminate pregnancy from sexual violence is good. The child has the right to live but pregnancy from sexual violence is different from regular pregnancy or pregnancy of young ladies who use their bodies or sex as business.*

37 year-old woman, married, who had terminated an SVRP.

*I knew I sinned. I knew what I did was illegal and I could be arrested if the information was disclosed…According to me to terminate a pregnancy is too bad because you kill and it’s a sin. It’s a loss since we don’t know what the child could become in the future.*

40 year-old woman, married, who had terminated an SVRP.

*As I didn’t have any shelter and I couldn’t shoulder responsibility for the child in case I delivered I decided to perform termination…To terminate a pregnancy is too bad because of severe aftermaths. One should not wish it. My main concern is that I have killed a fetus and lost dignity.*

28-year-old woman, not married, with two experiences of an SVRP (raising child from first SVRP, terminated most recent SVRP).

## Discussion

The qualitative findings from this study reveal complex decision-making processes related to SVRPs and highlight a myriad of factors that influenced decision-making among a sample of women with SVRPs in conflict-affected eastern DRC. The inclusion of women who continued and women who terminated the pregnancies allows for further dimension to the dialogue on decision-making in the context of SVRPs in conflict settings. Attitudes toward and beliefs about the SVRPs among women in both study groups influenced the decision-making processes related to the pregnancy.

Women considered the circumstances of sexual violence and the biologic, ethnic and social identities of the fetus and/or future child in relation to the perpetrator when making decisions related to SVRPs. The circumstances related to sexual violence were described as predominant influences for women who terminated an SVRP, similar to previous research on SVRPs [11, 13]. In both study subgroups, women expressed concerns related to the biologic and/or ethnic identity of the fetus and concerns related to social integration of the future child, especially given that the majority of perpetrators described in the interviews were armed combatants, usually of a different ethnic group. As noted in other studies on children born from SVRPs, the social identity of children is an important consideration as the children are often part of a hidden and marginalized population and may experience rejection from their families and communities [4, 31, 32]. Literature on children born from SVRPs describes how some children may know of their biologic father’s background, whereas other children may not have information about their biologic origin [31]. This may impact children born from SVRPs because certain social customs may follow a biologic lineage, such as naming of children or inheritance rights, [33] or access to social resources. Our data suggest that considerations of social identity are important influences on decision-making related to SVRPS and that these data could inform how future programs and policies address social integration of children born from these pregnancies.

The attitudes of spouses, families, and/or community members were also described as important influences on decision-making among the study population. Previously published data from this study noted that these attitudes impacted disclosure patterns among women with SVRPs [23]. In many cases, although not all, a supportive response from a spouse or family member may have influenced the woman to continue with the pregnancy. However, many women in our study reported negative reactions from their spouses and families, with some describing feeling forced to terminate the pregnancy to preserve marital and family relations. Women in the termination subgroup commonly described social stigmatization and rejection as predominant influences on decision-making. Previous research on sexual violence and SVRPs in conflict-affected eastern DRC revealed that survivors of sexual violence, especially women with SVRPs, experience social stigmatization, exclusion, and rejection; [20, 34–36] and our qualitative data support that perceived social consequences also influenced decision-making related to the SVRPs and should be considered when supporting women with SVRPs.

Religious and moral considerations related to pregnancy continuation and pregnancy termination were predominant themes in the qualitative analysis and influenced decision-making as noted in other studies [37, 38]. It is evident from our data that women were contemplating their pregnancies at the juncture of social and religious norms and the reality of sexual violence in a conflict-affected setting. As reported in other studies, restrictive social and religious norms may influence decision-making and disclosure of pregnancy termination [39], especially in settings with restricted access to termination services [40]. Furthermore, prior research revealed that women treating pregnancy termination as a moral issue were more likely to continue their pregnancies while women treating it as a personal issue were more likely to seek pregnancy termination [41]. The religious and moral contexts within which women with SVRPs consider their options should be taken into account when designing comprehensive reproductive health programs and policies.

The data highlight the power of religious beliefs over the autonomy of decision-making and women’s agency, even in the context of pregnancies conceived as a result of sexual violence. Among women who identified as Catholic, there were frequent references to God in the context of decision-making; reporting a fear of God to terminate the pregnancy or that the pregnancy was God’s will and should be continued. Some of the attitudes expressed in the interviews were similar to those noted in a study of religious perspectives on pregnancy termination [38]. One expert noted that according to the Catholic Church, the rights of human beings should not depend on the circumstances of their conception, such that a child conceived from sexual violence must be respected as any other child [38]. The study by Stephens et al. further articulated that according to Catholic understanding, taking a child’s life as a response to the crime of the father is not an option and that carrying a pregnancy conceived from violence would be an assertion of the woman’s strength and dignity and an assertion of the value of the child [38]. While the interview data echoed some of these perspectives, there were women who reported termination of the pregnancy in order to ensure their future well-being and who expressed that the termination of SVRPs should be considered differently than the termination of other pregnancies, which has important programming and policy implications.

Finally, within a reproductive justice framework, use of the term “decision-making” itself implies there are choices and options; which, in the context of SVRPs implies that a survivor of sexual violence should have access to comprehensive reproductive health services, including emergency contraception to prevent pregnancy, pregnancy termination, pregnancy care, and adoption services. However, in eastern DRC, pregnancy termination is restrictive, permissible only to save the life of a woman; thus, women may seek services through unskilled providers and/or use unsafe methods of termination, as noted in other studies and in our previous quantitative and qualitative assessments of data from the termination group [15, 17, 18, 22]. Little evidence exists on termination of SVRPs in other conflict-affected areas [42–44] and we believe that some of the findings presented here may have relevance to other conflict and post-conflict areas where comprehensive reproductive health care is limited and pregnancy termination laws are restrictive. It is notable that almost no women described an influence of a trained medical provider in their decision-making. This could be due to restrictive laws, poor access to reproductive health services, knowledge gaps among women or providers, or other unknown factors. Additionally, only two participants (both from the termination group) referenced the legality of pregnancy termination in DRC. While our study did not extensively assess attitudes towards laws pertaining to pregnancy termination or pregnancy care among women with SVRPs, future research should explore these areas for which there is little empiric data [45], especially in the setting of complex laws pertaining to pregnancy termination [15, 18]. Adoption, as a decision-making option in the context of SVRPs, was not directly assessed in this study and was not spontaneously disclosed in the interviews. Furthermore, the study criteria for the parenting group only included women who were currently caring for the child. However, adoption has been identified as an important aspect of dialogue on SVRPs and could be addressed in future research and programming related to SVRPs [4].

### Limitations

While women were initially recruited using RDS, the qualitative results represent a systematic convenience sample of participants; therefore, the results may not be generalizable. There are potential sources of biases, including interviewer bias, social desirability bias, and recall bias. Despite comprehensive interviewer training, it is possible that the interviewers influenced the interview with their own attitudes and beliefs about SVRPs, socially acceptable decisions, and complex feelings about pregnancy termination. Participants were asked questions about sensitive issues, and it is possible that their responses may have been influenced by a social desirability bias. Women were asked about decision-making in relationship to the SVRP, in some cases years after the event; therefore, there is the potential for limitations in recall due to time or due to the trauma of the event. However, the experience of sexual violence and a resultant SVRP is likely to be recalled as well as the decision-making process around this event. Additional biases could be introduced through the processes of translation, coding and analysis; however, the translator and investigators had prior expertise and training. The interviews were not audio-recorded in order to protect the participants and the findings rely on notes taken at the time of the interview rather than complete transcripts.

## Conclusion

This qualitative study provides insight into women’s experiences and decision-making regarding SVRPs in a conflict-affected setting. It provides perspectives from women who were raising children conceived from SVRPs and women who terminated SVRPs, which allows for greater dimension to the dialogue. Findings from both study groups reveal the complexities of decision-making related to SVPRs, the emotional reasoning, and the moral, religious, and social dimensions of the decision-making processes. The findings highlight the importance of considering the contexts and frameworks within which women with SVRPs process their circumstances and call attention to the importance of ensuring informed decision-making, including access to comprehensive reproductive health services, especially in conflict-affected settings.

## Abbreviations

DRC: Democratic Republic of the Congo
RDS: Respondent-driven sampling
SVRP: Sexual violence-related pregnancy
